# *C. elegans* Gene Regulatory Alleles and Reporter Bashing Studies

**DOI:** 10.17912/micropub.biology.000709

**Published:** 2023-01-20

**Authors:** Jonathan J Froehlich, Nikolaus Rajewsky

**Affiliations:** 1 Systems Biology of Gene Regulatory Elements, Berlin Institute for Medical Systems Biology, Max Delbrück Center for Molecular Medicine in the Helmholtz Association, Hannoversche Str. 28, 10115 Berlin, Germany; 2 Current affiliation: Systems Epigenetics, Otto Warburg Laboratories, Max Planck Institute for Molecular Genetics, 14195 Berlin, Germany

## Abstract

Gene regulation has been studied in *C. elegans* for over 30 years. In this analysis of 102 publications, we find that most transcriptional cis-regulatory elements are located within 5,000 bp of the transcription start site. Over 75% of studies conclude that transcriptional elements and 5′UTRs activate-, while 3′UTRs repress gene expression. While gene regulatory mutations make up less than 0.8% of alleles in forward genetics screens, recent CRISPR-Cas approaches are increasing the number of tested mutations. This work provides a resource of known gene regulatory sequences in *C.elegans* .

**Figure 1. Characteristics of gene regulatory sequences across published non-coding alleles and reporter studies:**
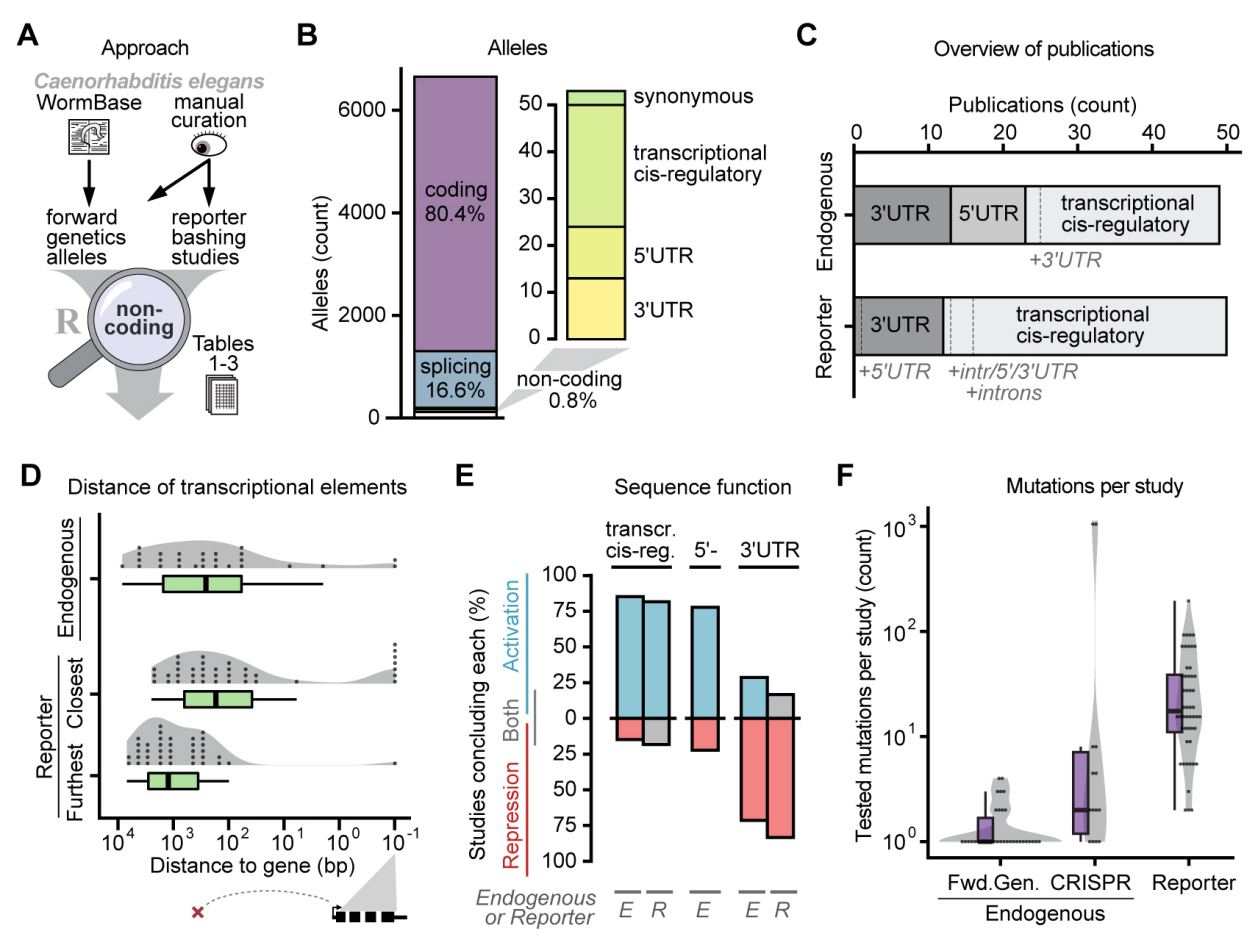
A) Overview of the approach. B) All alleles categorized by their impact on the host gene, with a zoom-in on non-coding alleles. C) Publications that describe endogenous mutations using forward genetics or CRISPR-Cas (“Endogenous”) and publications using reporter constructs (“Reporter”), categorized by the studied regions. D) Approximate distance of transcriptional gene regulatory elements to their target gene. For reporter studies, that often tested many variants, only the furthest and closest was extracted from the literature. E) Percentage of publications that concluded each of the inferred native sequence activities (activating, repressive, both) for the three different categories of gene regulatory regions. “Both” indicates that a study found both, activating and repressive elements. F) Tested mutations per study, categorized by technique.

## Description

Gene regulation is a vital process for multicellular organisms but identifying functional regulatory sequences and mechanisms can be challenging. In *C. elegans* , forward genetics can identify endogenous mutations (“alleles”) that disrupt a physiological process, and thus define functional sequences in an unbiased manner (Brenner 1974; Trent, Wood, and Horvitz 1988; Desai et al. 1988; Barton, Schedl, and Kimble 1987). CRISPR-based genome editing can be used to test endogenous sequences for function and physiological role (Dickinson and Goldstein 2016; Vicencio and Cerón 2021). Systematic testing of non-coding DNA in reporter assays (e.g., “reporter bashing”) can identify functional sequences but doesn’t allow direct examination of physiological function (Aamodt, Chung, and McGhee 1991; Didiano and Hobert 2006; Boulin, Etchberger, and Hobert 2006; Nance and Frøkjær-Jensen 2019).

Our goal was to summarize gene regulatory sequence studies and their findings. To do this, we used an R script to process published data, transcriptome annotation, and results from a literature search, and create main plots ( **Figure 1A** ). First, we downloaded a list of published mutations from WormBase (“classical alleles”, release WS284) (Harris et al. 2020). Of these, 82% were point mutations, the characteristic outcome of forward genetics screens using chemical treatment. We then curated all candidate non-coding alleles using primary literature and transcriptome annotation. Additionally, we catalogued reporter bashing publications. For both datasets, we gathered information about regulatory mechanisms, functions, phenotypes, distance to target genes, and more. The annotated non-coding alleles and reporter studies are listed in Tables 1-3 ( **Extended Data** ).

Few publications described their region of interest as “enhancer” while others did not use either the terms “promoter” and “enhancer”. To avoid confusion regarding the definition of these terms, we grouped all transcriptional gene regulatory sequences and refer to them as “transcriptional cis-regulatory”.

Out of all 7,046 *C. elegans* alleles, 80.4% directly changed protein sequence, 16.6% affected splicing, and only 0.8% (53 / 7,046) were non-coding ( **Figure 1B** ). Coding alleles primarily included missense mutations (47.4%) and stop-codon gains (27.7%), with the remaining alleles (5.3%) distributed across 8 more categories (e.g., start- or stop lost, frameshift, in-frame deletion or -insertion, transcript ablation). Splicing alleles disrupted splice acceptor (10.8%) or splice donor (4.6%) and included a few alleles of unclear consequence (1.2%). Future CRISPR-based studies may help us understand if the rarity of non-coding alleles reflects physiological function of regulatory regions, sequence robustness to point mutations, or experimental biases in historical screens.

The 53 non-coding alleles, disrupted by decreasing order of frequency: transcriptional cis-regulatory regions, followed by 3′UTRs, and 5′UTRs ( **Figure 1B** ). We did not include alleles of non-coding RNAs here (e.g., miRNAs) (Lee, Feinbaum, and Ambros 1993; Reinhart et al. 2000). However, we included synonymous mutations as “non-coding” since these may not directly change protein sequence and could affect gene regulation. Out of 53 non-coding alleles, there were only 3 synonymous mutations. Two of these created an ectopic splice donor and therefore changed protein sequence, while the mechanism for the last was unknown ( **Table 1, Extended Data** ). So far, we focused on alleles from forward genetics screens, to compare the relative importance of different genomic regions. When we included studies using CRISPR-based genome editing, the dataset contained 49 publications, and we also catalogued 50 representative reporter bashing studies ( **Figure 1C** ). More than half of publications clarified the underlying mechanisms ( **Tables 2 and 3, Extended Data** ). We then conducted a more detailed analysis of sequence features across the datasets.

We determined the typical location of transcriptional regulation. Transcriptional cis-regulatory elements were typically located between 0 and 5,000 bp (95-percentile) from the transcription start site, with most occurring between approximately 900 and 1,500 bp (95% confidence interval). The furthest element was recorded at around 8,500 bp and the closest elements were located within introns at 0 bp ( **Figure 1D** ). In forward genetics screens, 2 out of 20 (10%) alleles were in an intron or downstream of the gene.

We analyzed the gene regulatory activity of the studied regions. For this we assigned an activating function when mutations resulted in downregulation or a repressive function when mutations resulted in upregulation. Over 75% of studies found that transcriptional cis-regulatory regions and 5′UTRs contained activating sequences, while 3′UTRs contained repressive sequences ( **Figure 1E** ). This analysis may be more extreme when weighing by the number of examined mutations or genes. On the other hand, some assay readouts may be biased and not detect up- or downregulation equally well. For 3′UTRs the “activating” category also contained ectopic nonsense-mediated decay mutations which don’t point to a native activating element, leaving only polyadenylation sites as true activating elements in 3′UTRs.

Finally, we looked at the throughput of experiments studying gene-regulatory sequences ( **Figure 1F** ). All reporter publications examined 1,625 mutations, among which the largest tested 195 mutations (Stefanakis, Carrera, and Hobert 2015). Endogenous publications tested only 87 mutations in total, with forward screens 1.4 on average. In contrast, recent multiplexed and parallelized CRISPR-Cas approaches reached over 1,000 tested endogenous mutations per study (Yang, Schwartz, and McJunkin 2020; Froehlich et al. 2021). It is likely that similar approaches will expand the scale of future gene regulatory studies in *C. elegans* .

## Methods

The R script to reproduce analyses and plots and a Rmarkdown html notebook can be found at: https://github.com/jonathanfroehlich/Celegans_GeneRegAlleles_and_RepBashStudies. The script performs the following main steps: 1. Downloads “classical alleles” and transcriptome/genome annotation from WormBase; 2. Cleans up overlapping transcriptome features to remove overlaps conservatively (e.g., 3′UTR sequence that overlaps with coding sequence); 3. Categorizes each classical allele by its overlap with genomic feature (e.g., coding, synonymous, 5′-/3′UTR, intergenic); 4. Exports a spreadsheet with this information together with the original WormBase annotation; 5. This table then has to be populated manually for example in a common spreadsheet software (we provide the annotated table in our code repository). Our criteria for manual curation are described below; 6. Exports separate .bed files of alleles according to their overlap with genomic feature; 7. Loads the manually populated spreadsheet; 8. Exports a .pdf with the plots shown in this publication, plus additional plots; 9. Exports individual browser shots of each allele together with the transcriptome annotation to help in further curation. The script uses mainly: R, Rstudio, data.table, GenomicRanges, rtracklayer, Gviz (R Core Team 2022; RStudio Team 2015; Lawrence et al. 2013; Dowle and Srinivasan 2022; Lawrence, Gentleman, and Carey 2009; Hahne and Ivanek 2016). We accessed WormBase release WS284, August 2022 (Harris et al. 2020).

During our manual curation of non-coding alleles we conservatively excluded those that were likely not truly non-coding or not gene regulatory, as following: alleles that were found to change protein sequence in any gene isoform, either by the original publication or by us after inspecting genome browser and annotated isoforms; or alleles that were not isolated due to an effect on phenotype or function (passenger mutations); or alleles with insufficient information on any functional or phenotypic consequence and no associated publication. Using these criteria, we excluded 32 from 35 potential synonymous alleles, and 26 from 76 potential alleles of transcriptional- (promoter/enhancer) and post-transcriptional (5′-/3′UTRs) cis-regulatory alleles. With a thorough literature search we also found 21 publications that each described various numbers of gene-regulatory alleles not registered in WormBases classical alleles. Tables 1 and 2 (Extended Data) contain the additionally catalogued alleles and publications, as well as the excluded alleles with the reason for their exclusion.

To distinguish different transcriptional regulatory alleles, we assigned sequences within +100 bp of the transcription start site as “core promoter” (Grishkevich, Hashimshony, and Yanai 2011), elements that were described as “enhancer” in the original publication as “enhancer” and the remaining elements simply as “promoter”. This information can be found in the Extended Data. As mentioned in the main text we collectively call all these elements simply “transcriptional cis-regulatory”, to avoid confusion arising from the definition of enhancers and promoters.

We also collected 50 publications that used systematic reporter bashing to find gene regulatory sequences, these publications can be found in Table 3 (Extended Data). Here we did not distinguish “core promoters”, “promoters” or “enhancers” and used one category “transcriptional cis-regulatory”. This list of publications was likely incomplete but was useful to complement the following analyses.

We estimated the distance of functional gene regulatory sequences to their target genes visually using the available figure and scale bar, or in Wormbase JBrowse 2, often by rounding to 50s and 100s; or several 100s for the very distant elements. These numbers are thus only rough estimates. For reporter studies, which often tested many sequences (and in some cases also several genes), we only wrote down the closest and furthest active sequence per publication. These numbers are therefore incomplete but give an upper and lower limit.

We collected the gene regulatory effect of a mutation (either “down” or “up”) for both datasets (alleles and reporters). Here we considered gain of expression in extra cells, tissues, or stages as “up” and reduction or complete loss as “down”. In some cases, the gene regulatory effect was not measured directly but inferred by phenotype in the primary publications. The allele table (Table 2) also contains a column that distinguishes “null” from “reduced” whenever that information was available, but for the analysis of sequence activity both cases were assigned as “down”.

## Extended Data

Description: Manually curated gene regulatory non-coding alleles and reporter bashing studies. Resource Type: Dataset. DOI: 10.22002/68z70-27896
